# The prognostic impact of net ultrafiltration intensity in critically ill patients receiving continuous renal replacement therapy: a multivariable and propensity-matched analysis

**DOI:** 10.1080/0886022X.2025.2537810

**Published:** 2025-07-29

**Authors:** Lu Jin, Peiyun Li, Youli Tang, Wanhong Yin, Fang Wang, Ling Zhang

**Affiliations:** a Division of Nephrology, Kidney Research Institute, West China Hospital of Sichuan University, Chengdu, China; b Department of Critical Care Medicine, Wenjiang District People’s Hospital, Chengdu, China; c Department of Critical Care Medicine, West China Hospital of Sichuan University, Chengdu, China

**Keywords:** Net ultrafiltration, continuous renal replacement therapy, acute kidney injury, fluid overload

## Abstract

**Objectives:**

Net ultrafiltration (UF^net^) is widely used for fluid management during continuous renal replacement therapy (CRRT) for critically ill patients over extended periods. Despite widespread use, the relationship between UF^net^ intensity and clinical outcomes, particularly mortality, remains controversial.

**Methods:**

This retrospective observational study examined critically ill patients undergoing CRRT for more than 72 h from January 2021 to September 2023. Patients were stratified by their UF^net^ intensity during the initial 72 h of CRRT into low (<1.01 mL/kg/h), moderate (1.01–1.75 mL/kg/h), and high (>1.75 mL/kg/h) groups. The primary outcome was 28-day mortality. Kaplan–Meier’s survival curves with log-rank tests, Cox proportional hazards models, and propensity score matching were employed to assess the association between UF^net^ intensity and mortality.

**Results:**

A total of 683 patients were included. Compared with the moderate UF^net^ intensity, the low UF^net^ intensity (adjusted hazard ratio (HR) 1.54, 95%CI 1.24–1.91, *p* = .024) and high UF^net^ intensity (adjusted HR 1.27, 95%CI 1.03–1.57, *p* < .001) were associated with higher 28-day mortality. Sensitivity analyses showed similar trends for 60-day and 90-day mortality. Subgroup analyses based on admission diagnosis did not reveal significant differences in the effect of UF^net^ intensity on mortality risk.

**Conclusions:**

UF^net^ intensity between 1.01 and 1.75 mL/kg/h during the first 72 h of CRRT was associated with lower 28-day mortality compared to both lower and higher UF^net^ intensities. However, future studies are needed to better define optimal UF^net^ thresholds in multicenter ICU cohorts.

## Graphical Abstract

**Figure:**
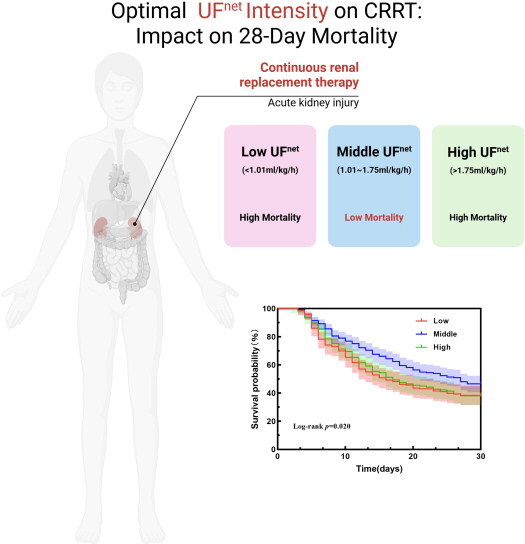

## 1. Introduction

Acute kidney injury (AKI), a condition common among patients in intensive care units (ICUs), often requires continuous renal replacement therapy (CRRT) [1,2]. Fluid overload (FO), frequently associated with AKI, has been identified as an independent predictor of increased mortality [3,4]. Timely adjustment of the net ultrafiltration (UF^net^) intensity is crucial for accurate volume management and addressing patients’ hemodynamic and fluid balance needs [5,6]. However, the optimal intensity of fluid removal remains a subject of ongoing debate [7,8], with current guidelines offering limited guidance on the precise UF^net^ intensity that maximizes patient survival [5,9,10].

Previous studies have investigated the impact of varying UF^net^ intensity on clinical outcomes [11–16]. One study revealed that a higher UF^net^ intensity (>1.75 mL/kg/h), particularly during the initial 48 h, was associated with a significant rise in 28-day mortality [15]. Another study suggested a positive correlation between more aggressive fluid removal and improved short-term survival, though results varied across different patient cohorts [16]. Additionally, observational research indicated a “J”-shaped relationship between UF^net^ intensity and mortality in critically ill patients [13]. Notably, these studies were not conducted in Asia. Given the variability in patient responses to fluid management, further investigation is needed to develop evidence-based guidelines for UF^net^ intensity in critically ill patients with AKI receiving CRRT.

We examined the effect of UF^net^ intensity on mortality in critically ill adults receiving CRRT. Specifically, we assessed whether moderate UF^net^ intensity (1.01–1.75 mL/kg/h) improved clinical outcomes in this contemporary cohort.

## 2. Methods

### 2.1. Study design

We conducted a single-center retrospective cohort study of patients treated with CRRT from January 2021 to September 2023 at West China Hospital of Sichuan University. Because of the non-interventional nature of the study, the requirement for informed consent was waived by the local ethics committees (ethical approval: the Ethics Review Committee of West China Hospital of Sichuan University; Project number: 2023.1967).

### 2.2. Study population

Patients included in this analysis were those who (1) were more than 18 years of age, diagnosed with AKI, and started CRRT within 14 days of ICU admission; and (2) had a CRRT duration time of over 72 h. CRRT indications included severe AKI (KDIGO stage 2/3), refractory hypervolemia, or metabolic acidosis. Patients who met the following criteria were excluded: (1) patients who died within 72 h of ICU admission; (2) end-stage kidney disease (ESKD) patients; (3) patients who received other modalities of blood purification, such as intermittent hemodialysis (IHD) or plasma exchange (PE); and (4) patients with incomplete critical variables required to calculate UF^net^ intensity in the first 72 h of CRRT.

### 2.3. CRRT protocol

CRRT was performed using the Prismaflex^®^ device (Baxter, Chicago, IL) or the MultiFiltrate^®^ device (Fresenius Medical Care, Bad Homburg, Germany) in a post-dilution continuous veno-venous hemodiafiltration (CVVHDF) mode, with an approximate 1:1 dialysis-to-filtration ratio. Anticoagulation was tailored to the patient’s clinical condition, with options including regional citrate anticoagulation, low-molecular-weight heparin, nafamostat mesylate, or no anticoagulation. Blood flow protocols ranged from 120 to 200 mL/min, and effluent rates were set to 25–30 mL/kg/h, in alignment with the Kidney Disease: Improving Global Outcomes (KDIGO) guidelines [1].

### 2.4. Data extraction

We extracted data on demographic characteristics (age, sex, race, height, weight, body mass index (BMI), type of ICU, and ICU admission diagnosis), illness severity on the first day of admission (Acute Physiology and Chronic Health Evaluation (APACHE II) score), sequential organ failure assessment (SOFA) score prior to CRRT, Glasgow Coma Scale (GCS) score before CRRT, vasopressor requirements at CRRT initiation, laboratory tests, vital signs, and CRRT-related parameters. CRRT data included the ultrafiltration metrics, fluid balance in the 24 h preceding CRRT, the interval from ICU admission to CRRT initiation, and CRRT duration. Additionally, we collected the Vasoactive-Inotropic Score (VIS) at the 72 h of CRRT and clinical outcomes. Data were obtained from electronic databases and scanned medical records.

### 2.5. Definitions

Two independent adjudicators determined each patient’s ICU admission diagnosis, with a third adjudicator resolving disagreements if necessary. Sepsis was defined as life-threatening organ dysfunction due to dysregulation of the host response caused by infection, identified by an acute change in the total SOFA score ≥2 points following the infection [17,18]. Fluid overload [19] was calculated as follows: FO (%) = [total daily fluid intake (mL) − total daily fluid output (mL)]/baseline weight × 100%. In this study, fluid balance was calculated for the 24 h preceding CRRT; for durations less than 24 h, calculations were based on actual time. VIS quantified vasoactive and inotropic drug dosage, reflecting both cardiac function and therapy intensity in critically ill patients [20]. Kidney recovery was defined as survival without the need for CRRT during the follow-up period.

### 2.6. Exposure

UF^net^ intensity was calculated by the following formula [21]:

$${\text{UF}}^{\text{net}}\text{ intensity }(\text{mL/kg/h})=\frac{\text{total }{\text{UF}}^{\text{net}}\text{ volume }(\text{mL})}{\text{hospital weight admission }(\text{kg})\times \text{CRRT duration }(h)}$$

### 2.7. Study outcome

The primary outcome was 28-day mortality. Secondary outcomes included the duration of mechanical ventilation, kidney recovery, length of ICU and hospital stay, as well as ICU, hospital, 60-day, and 90-day mortality.

### 2.8. Statistical analysis

**Figure 1.**
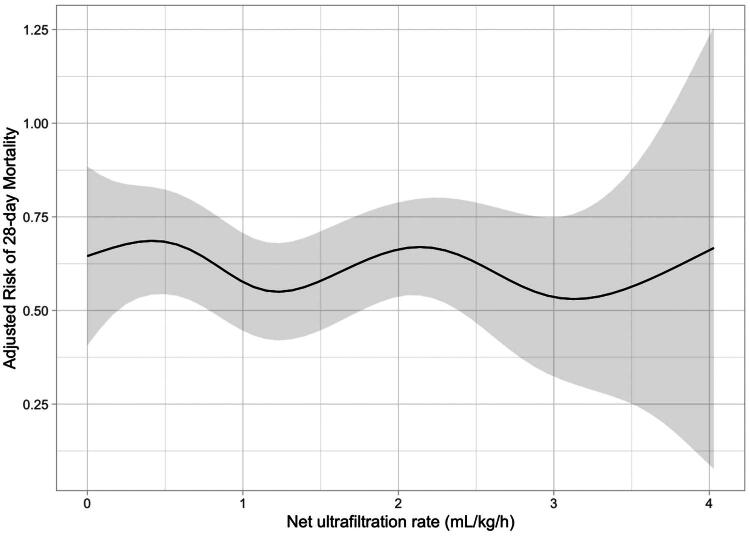
The association of UF^net^ intensity and risk of 28-day mortality. The association was plotted using a multivariate generalized additive linear model, which accounts for age, gender, body mass index, diagnosis, baseline serum creatinine, mean arterial pressure before CRRT, lactate before CRRT, oxygenation index before CRRT, sequential organ failure assessment score before CRRT, Acute Physiology and Chronic Health Evaluation II score before CRRT, oliguria before CRRT, the percent of patients with FO >5% before CRRT, and cumulative fluid overload percent in the first 24 h of CRRT.

First, we confirmed a non-linear association between UF^net^ intensity and 28-day mortality using a multivariate generalized additive model (Figure 1). Based on prior studies [12,15], we stratified UF^net^ intensity in the first 72 h into three categories: low (<1.01 mL/kg/h), middle (1.01–1.75 mL/kg/h), and high (>1.75 mL/kg/h). Kaplan–Meier’s survival curves with log-rank tests were employed to compare mortality across these groups. Univariable and multivariable Cox proportional hazards models were used to analyze the association between UF^net^ intensity (as a categorical variable) and mortality. Continuous confounding variables in the model included age (years), sex (% male), weight (kg), baseline serum creatinine (mg/dL), MAP (mean arterial pressure, mmHg), APACHE II score (per 10%), with or without FO >5% before CRRT, ICU admission diagnosis, and the ICU-to-CRRT initiation interval. Additionally, UF^net^ intensity in the first 24 and 48 h was grouped and analyzed similarly. Patients with missing variables relevant to UF^net^ calculation were excluded from the analysis. While advanced imputation methods, such as multiple imputation by chained equations (MICEs), were considered, a complete-case analysis was chosen due to the small proportion of missing data (23 patients) to ensure the integrity and directness of the primary analysis. To control for potential confounding, we performed propensity score matching (PSM) [22] using a multivariable logistic regression model. Matching was performed in a 1:1:1 ratio using greedy nearest-neighbor matching with a caliper width of 0.2 standard deviations of the propensity score.

For subgroup analyses, we stratified patients by ICU admission diagnosis and applied Cox proportional hazards models to evaluate the association between UF^net^ intensity and mortality risk within these subgroups’ diagnostic categories.

We summarized categorical variables as counts and percentages, comparing them with Chi-square tests. For continuous variables, we reported medians and interquartile ranges (IQRs; 25th–75th percentiles) and used Wilcoxon’s rank-sum tests for comparisons. All hypothesis tests were two-tailed, and we considered *p* values <.05 statistically significant. We conducted all analyses using R software (version 4.4.0) (R Foundation for Statistical Computing, Vienna, Austria).

## 3. Results

### 3.1. Study population

**Figure 2.**
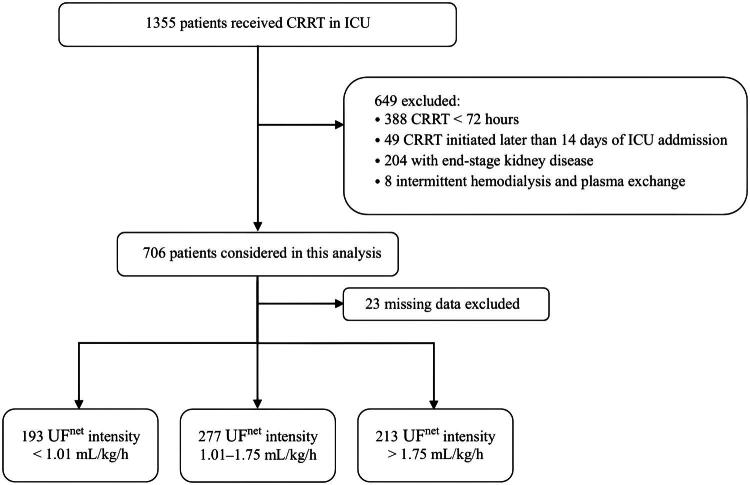
Flowchart of study selection.

Between January 2021 and September 2023, a total of 1,355 critically ill patients were admitted to the ICU and treated with CRRT. Of these, 57 patients were excluded due to receiving IHD or PE or initiation of CRRT beyond 14 days of ICU admission. Additionally, 388 patients with a CRRT duration of less than 72 h were excluded. Twenty-three patients with missing data for variables of interest were also excluded, finally leaving 683 patients included in the study. Among included patients, 193 (28.3%) had UF^net^ intensity of <1.01 mL/kg/h, 277 (40.6%) had UF^net^ intensity of 1.01–1.75 mL/kg/h, and 213 (31.2%) had UF^net^ intensity of >1.75 mL/kg/h (Figure 2).

The baseline characteristics of the study population according to UF^net^ intensity are summarized in Table 1. The median UF^net^ intensity was 1.37 (IQR 0.94–1.89) mL/kg/h. Patients with UF^net^ intensity >1.75 mL/kg/h were older, more frequently female, and had lower body weight, higher N-terminal pro-brain natriuretic peptide (NT-proBNP) levels, and lower platelet counts prior to CRRT. Patients in the 1.01–1.75 mL/kg/h UF^net^ intensity group had lower pre-admission creatinine levels and were more likely to use vasopressors before CRRT. No significant differences were observed across UF^net^ intensity groups in disease severity scores (APACHE II or SOFA), key baseline characteristics (e.g., sepsis diagnosis), urine output in the 24 h before CRRT, or the percent of patients with FO >5% before CRRT. However, patients in the <1.01 mL/kg/h group had higher lactate levels prior to CRRT initiation.

**Table 1.** Baseline characteristics of each UF^net^ intensity group before CRRT.

Characteristics	UF^net^ intensity category (72 h)			*p* Value
	<1.01 mL/kg/h (*n* = 193)	1.01–1.75 mL/kg/h (*n* = 277)	>1.75 mL/kg/h (*n* = 213)	
Age (years)	54 (43–68)	55 (44–68)	58 (48–71)	.037
Male gender, *n* (%)	152 (78.8%)	220 (79.4%)	138 (64.8%)	<.001
Weight (kg)	70 (60–80)	70 (60–80)	60 (50–70)	<.001
BMI (kg/m^2^)	25.1 (22.2–28.0)	25.1 (22.4–27.7)	22.3 (19.2–24.9)	<.001
Pre-admission kidney function				
Creatinine (mmol/L)	224 (152–362)	281 (182–401)	245 (154–377)	.005
eGFR (mL/min/1.73 m^2^)	23.9 (15.0–40.9)	18.8 (12.8–33.2)	19.4 (12.7–40.3)	.006
ICU type, *n* (%)				.141
General surgery	18 (9.3)	21 (7.6)	20 (9.4)	
Cardiac and coronary	10 (5.2)	16 (5.8)	14 (6.6)	
Cardiovascular surgery	34 (17.6)	31 (11.2)	18 (8.5)	
Neurological	6 (3.1)	8 (2.9)	9 (4.2)	
Medical	9 (4.7)	20 (7.2)	23 (10.8)	
Miscellaneous	116 (60.1)	181 (65.3)	129 (60.6)	
ICU admission diagnosis, *n* (%)				.979
Cardiovascular	13 (6.7)	23 (8.3)	19 (8.9)	
Cardiothoracic surgery	33 (17.1)	32 (11.6)	25 (11.7)	
Neurology	11 (5.7)	12 (4.3)	12 (5.6)	
Pneumonia	6 (3.1)	11 (4.0)	9 (4.2)	
Respiratory-not pneumonia	4 (2.1)	7 (2.5)	4 (1.9)	
Hematology	2 (1.0)	4 (1.0)	2 (0.9)	
Digestive	52 (26.9)	83 (30.0)	62 (29.1)	
Sepsis	52 (26.9)	76 (27.4)	52 (24.4)	
Trauma	13 (6.7)	19 (6.9)	15 (7.0)	
Other medical	7 (3.6)	10 (3.6)	13 (6.1)	
Before CRRT initiation				
Intervals from ICU admission to CRRT (days)	1.0 (0.4–3.0)	0.9 (0.3–4.0)	1.3 (0.3–4.0)	.417
Need of vasopressor, *n* (%)	109 (56.5)	190 (69.6)	132 (62.0)	.025
Fluid balance in the 24 h before CRRT (mL)	1,800 ((894–2,999.3)	1,856 (995–2,737)	1,506.5 (748–2,908)	.375
Fluid overload >5%, *n* (%)	34 (17.6)	49 (17.7)	41 (19.2)	.883
Urine output at 24 h (mL)	650 (265–1,563)	575.5 (147.5–1,311.3)	490 (150–1,085)	.052
Oliguria, *n* (%)	69 (35.8)	115 (41.5)	90 (42.3)	.339
APACHE II score	21 (14–27)	22 (16–28)	21 (15–27)	.237
SOFA score	16 (14–18)	17 (15–19)	17 (15–18)	.232
GCS score	6 (4–7)	5 (4–8)	5 (4–7)	.987
Laboratory before CRRT				
Lactate (mmol/L)	2.3 (1.5–4.3)	2 (1.4–3.4)	2 (1.3–3.1)	.009
PaO_2_/FiO_2_	234.7 (144.5–327.6)	211.3 (146.2–303.3)	229 (156.2–312.1)	.428
MAP (mmHg)	84.3 (76–95.3)	85 (75.7–94.7)	86 (75.3–97)	.859
NT-proBNP	3,326 (993–9,412)	4,198 (1,260.3–11,262.8)	5423.5 (1,817.8–15,215)	.002
TBIL	24 (13.2–52.8)	21.1 (10.8–49.4)	20.3 (11.6–51.4)	.319
PLT	94 (53–167)	95 (53–154)	77 (43–139)	.032

UF^net^: net ultrafiltration; CRRT: continuous renal replacement therapy; BMI: body mass index; eGFR: estimated glomerular filtration rate; ICU: intensive care unit; APACHE II: Acute Physiology and Chronic Health Evaluation; SOFA: sequential organ failure assessment; MAP: mean arterial pressure; NT-proBNP: N-terminal pro-brain natriuretic peptide; TBIL: total bilirubin; PLT: platelet.

As shown in Table 2, patients with UF^net^ intensity >1.75 mL/kg/h underwent a longer duration of CRRT. Those in the <1.01 mL/kg/h group exhibited higher VIS scores after 72 h of CRRT. Patients in the 1.01–1.75 mL/kg/h group had increased length of hospital and ICU stay. There were no significant differences among the groups in the duration of mechanical ventilation, kidney recovery, or mortality.

**Table 2.** Characteristics of the therapy and clinical outcomes.

Characteristics	UF^net^ intensity category (72 h)			*p* Value
	<1.01 mL/kg/h (*n* = 193)	1.01–1.75 mL/kg/h (*n* = 277)	>1.75 mL/kg/h (*n* = 213)	
CRRT duration (hours)	127 (102.5–145)	133 (110–147)	138 (115–149)	.016
Hemodynamic				
VIS at the 72 h of CRRT	4.95 (0–44.9)	5 (0–25.2)	0.1 (0–14.9)	.003
UF^net^ intensity				
Hourly (mL/h)	46.8 (29.8–61.8)	92.2 (76.7–108.3)	134.8 (114.2–154.5)	<.001
Cumulative (mL)	7,317 (4,412–11,223)	13,079 (9,605–16,497)	16,984 (13,333–20,054)	<.001
Clinical outcomes				
Duration of mechanical ventilation (days)	12 (7–22)	15 (9–24)	14 (8–20)	.119
Kidney recovery, *n* (%)	53 (100)	90 (98.9)	48 (94.1)	.073
Length of stay (days)				
ICU	15 (10–26)	19 (12–30.3)	17 (11–26.3)	.010
Hospital	21 (13–33)	25 (16–43)	22 (15–38)	.004
Mortality, *n* (%)				
ICU	125 (35.2)	163 (58.8)	142 (66.7)	.171
Hospital	139 (72.0)	187 (67.5)	156 (73.2)	.337
28 days	119 (61.7)	148 (53.4)	132 (62.0)	.092
60 days	138 (71.5)	179 (64.6)	157 (73.7)	.073
90 days	140 (72.5)	186 (67.1)	162 (76.1)	.089

UF^net^: net ultrafiltration; CRRT: continuous renal replacement therapy; VIS: Vasoactive-Inotropic Score.

### 3.2. Associations between UF^net^ intensity and primary outcome

**Figure 3.**
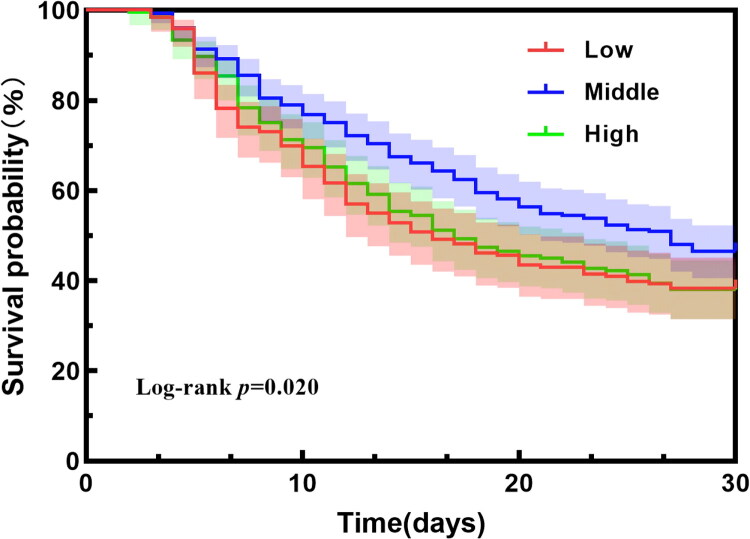
Kaplan–Meier’s survival plots with the log-rank test by different UF^net^ intensity categories in the first 72 h.

We used Kaplan–Meier’s survival curves with the log-rank test to examine the association between UF^net^ intensity in the first 72 h and 28-day mortality (Figure 3). Using a Cox proportional hazards model, we found that compared to UF^net^ intensity of 1.01–1.75 mL/kg/h, the low UF^net^ intensity group (<1.01 mL/kg/h) had a higher risk of 28-day mortality (unadjusted hazard ratio (HR) = 1.35, 95%CI: 1.06–1.72; *p* = .016). Similarly, the high UF^net^ intensity (>1.75 mL/kg/h) group also demonstrated an increased risk (unadjusted HR = 1.31, 95%CI: 1.03–1.65; *p* = .026) (Table 3, model 1). After adjusting for all confounding variables, both the low and high UF^net^ intensity showed significantly higher 28-day mortality risk (low: adjusted HR = 1.54, 95%CI: 1.24–1.91; *p* < .001; high: adjusted HR = 1.27, 95%CI: 1.03–1.57; *p* = .024) (Table 3, model 4). Further model details are provided in Supplementary Table S1.

**Table 3.** The association of UF^net^ intensity and 28-days mortality.

	Model 1		Model 2		Model 3		Model 4	
	HR (95%CI)	*p*	HR (95%CI)	*p*	HR (95%CI)	*p*	HR (95%CI)	*p*
UF^net^ intensity (mL/kg/h)								
>1.75 mL/kg/h	1.31 (1.03–1.65)	.026	1.14 (0.89–1.46)	.299	1.14 (0.88–1.47)	.322	1.27 (1.03–1.57)	.024
1.01–1.75 mL/kg/h	1.00 (Reference)	–	1.00 (reference)	–	1.00 (reference)	–	1.00 (reference)	–
<1.01 mL/kg/h	1.35 (1.06–1.72)	.016	1.57 (1.23–2.01)	<.001	1.46 (1.13–1.90)	.004	1.54 (1.24–1.91)	<.001

UF^net^: net ultrafiltration; HR: hazard ratio; CI: confidence interval; model 1: crude; model 2: adjust: age, sex, and weight; model 3: adjust: age, sex, weight, creatinine, Acute Physiology and Chronic Health Evaluation II score, map, fluid overload > 5%; model 4: adjust: age, sex, weight, creatinine, Acute Physiology and Chronic Health Evaluation II score, map, fluid overload >5%, diagnosis, interval from admission to CRRT. The bold values are used to flag results where *p* < 0.05.

### 3.3. Sensitivity analyses

We conducted additional analyses to assess the relationship between UF^net^ intensity in the first 72 h after CRRT initiation and 60-day and 90-day mortality, using Kaplan–Meier’s survival plots and the Cox proportional hazards model. After adjusting for confounders, patients with UF^net^ intensity of 1.01–1.75 mL/kg/h had a significantly lower mortality risk than those in the higher or lower UF^net^ intensity groups (Supplementary Figure S1; Tables S2 and S3).

We also analyzed the association between UF^net^ intensity during the first 24 and 48 h of CRRT and mortality. Dividing patients into three UF^net^ intensity categories based on the same thresholds, we found no statistically significant differences in survival across UF^net^ intensity groups in the first 24 or 48 h, as assessed by Kaplan–Meier’s survival plots with the log-rank test (Supplementary Figures S2 and S3).

We performed PSM using covariates identified as potential risk factors. These covariates included demographic characteristics (age, sex, weight, BMI), illness severity and comorbidities (e.g., ICU admission diagnosis, APACHE II score), and pre-CRRT laboratory values and vital signs (e.g., creatinine, platelet count, MAP, NT-proBNP). The matching yielded a cohort of 303 patients (101 per group), with baseline characteristics well balanced between groups post-matching. Kaplan–Meier’s survival analysis demonstrated that patients receiving a UF^net^ intensity of 1.01–1.75 mL/kg/h had significantly lower 28-day mortality (Supplementary Table S4 and Figure S4).

### 3.4. Analyses by admission diagnosis

Patient characteristics, clinical outcomes, and CRRT data stratified by admission diagnosis are provided in Supplementary Table S4. No significant differences in UF^net^ intensity were observed across admission diagnoses (Supplementary Figure S5). Patients with hematologic diagnoses had relatively higher median UF^net^ intensity of 1.67 mL/kg/h (IQR: 1.00–2.34), while those admitted for non-pneumonic respiratory conditions or cardiothoracic surgery had lower median UF^net^ intensity of 1.21 mL/kg/h (IQR: 0.84–1.58) and 1.32 mL/kg/h (IQR: 1.16–1.47), respectively. There were no significant differences in the impact of UF^net^ intensity on mortality risk across admission diagnoses (Supplementary Figure S4; Supplementary Table S5 and Figure S6).

## 4. Discussion

### 4.1. Key findings

We conducted a detailed analysis of UF^net^ intensity data from over 600 ICU patients treated between 2021 and 2023. Using electronic CRRT machine data, we evaluated UF^net^ intensity characteristics and their modulation across different timeframes. Our primary objective was to assess whether cutoff values established in previous international studies remain applicable to Asian populations.

Our findings revealed that UF^net^ intensity <1.01 mL/kg/h or >1.75 mL/kg/h was associated with increased mortality compared to a UF^net^ intensity of 1.01–1.75 mL/kg/h during the first 72 h of CRRT, even after adjusting for confounding factors such as age, weight, and illness severity at admission. Additionally, UF^net^ intensity >1.75 mL/kg/h was significantly associated with prolonged CRRT duration, while UF^net^ intensity of 1.01–1.75 mL/kg/h was linked to extended ICU and hospital stay.

### 4.2. Relationship to previous studies

Some studies have identified a J-shaped relationship between UF^net^ intensity and mortality, a pattern consistent with our study results. Secondary analyses of the RENAL trial, which corroborated our findings, also found reduced 90-day mortality for patients within the 1.01–1.75 mL/kg/h range [12]. Similarly, Wu et al. observed lower 28-day mortality in patients with UF^net^ intensity of 1.6–3.1 mL/kg/h compared to those with intensity >3.1 or <1.6 mL/kg/h, though their cutoff values differed significantly from those used in other studies, suggesting variability in the impact of UF^net^ intensity on mortality [13].

On the contrary, Naorungroj et al. identified an independent association between UF^net^ intensity >1.75 mL/kg/h and increased 28-day mortality compared to UF^net^ intensity <1.01 mL/kg/h [15]. Nonetheless, their study included a smaller sample and patients with ESKD. Similarly, this article did not support a significant association between higher UF^net^ intensity and greater hemodynamic instability, aligning with our findings. Sansom et al. also reported a link between higher UF^net^ intensity and increased ICU mortality, with subgroup analyses suggesting that the effect of UF^net^ intensity on mortality may vary by admission diagnosis [16]. As highlighted in multinational surveys [23,24], there is international variation in the prescription of UF^net^ intensity. The discrepancies observed across the studies may be attributed to differences in CRRT practices across regions.

### 4.3. Implications of study findings

Our analysis demonstrates that moderate UF^net^ intensity (1.01–1.75 mL/kg/h) during the initial 72 h of CRRT is associated with reduced mortality, consistent with prior studies describing a J-shaped mortality-UF^net^ relationship. Excessively high UF^net^ intensity (>1.75 mL/kg/h) may induce hemodynamic instability, while inadequate UF^net^ intensity (<1.01 mL/kg/h) exacerbates FO, both contributing to adverse outcomes. Elevated NT-proBNP levels in the high UF^net^ group suggest underlying hypervolemia, which may prompt clinicians to prescribe aggressive fluid removal, potentially destabilizing vulnerable patients.

Mechanistically, higher UF^net^ intensity may precipitate hemodynamic compromise, particularly in patients with undiagnosed diastolic dysfunction or reduced cardiac reserve, while also increasing risks of electrolyte imbalances (e.g., hypokalemia) and delayed renal recovery. Conversely, lower UF^net^ intensity perpetuates FO, worsening tissue oxygenation and intra-abdominal hypertension, as evidenced by elevated lactate levels in this subgroup. Although patients with moderate UF^net^ exhibited prolonged ICU and hospital stays, this likely reflects their improved survival rather than iatrogenic harm.

To exclude the influence of the severity of patients’ underlying diseases on mortality, we excluded patients with a CRRT duration of less than 72 h. However, our study found that UF^net^ intensity in the first 24 or 48 h of CRRT initiation did not significantly impact mortality. This finding suggests that while UF^net^ intensity reflects fluid balance during CRRT, managing fluid balance in critically ill patients requires consideration of additional factors such as fluid input from other medications and blood products, and fluid output through sweating and gastrointestinal losses. Other early-phase factors affecting mortality in CRRT still need further exploration.

### 4.4. Strengths and limitations

This study represents the first large-scale, population-based investigation in China examining the relationship between UF^net^ intensity and prognosis in patients undergoing CRRT. Our analysis explored the prognostic significance of UF^net^ intensity across various admission diagnoses and timeframes. With an extended follow-up period, we compared 28-day, 60-day, and 90-day mortality among different groups, further reinforcing the reliability and robustness of our findings.

However, several limitations should be acknowledged. First, as a single-center, retrospective observational study, establishing a causal relationship between UF^net^ intensity and mortality risk was challenging. Although the study population was drawn from a leading ICU medical center in western China, the design’s inherent limitations restrict the generalizability of these findings to other ICU populations. Second, while baseline characteristics of the grouped populations varied, the severity of illness did not show statistically significant differences. Despite adjusting for numerous risk factors and potential confounders, residual confounding may still have influenced the observed associations. Additionally, 23 patients were excluded due to missing critical variables (e.g., UF^net^-related parameters). Although sensitivity analyses indicated these exclusions did not materially affect the outcomes, the possibility of selection bias cannot be ruled out. Moreover, diuretic use and dosing were incompletely documented, as most patients discontinued diuretics after initiating CRRT. This limitation may have confounded the observed associations between UF^net^ intensity and mortality. Third, the limited sample size may have affected the robustness of our results. Although subgroup analyses based on admission diagnoses were conducted, our findings differed from those reported in Sansom et al.’s research [16], potentially indicating false-negative outcomes. Fourth, FO >5% was not identified as a prognostic factor in our study. This result may stem from the inability to accurately assess true fluid balance in most patients prior to CRRT initiation. Many patients were transferred directly from external hospitals, often without documented fluid balance or data on diuretic use at admission. In our cohort, fewer than 20% of patients had FO >5% before CRRT, a significantly lower proportion than reported in other studies. Consequently, reliable statistical analysis of fluid balance and its relationship with prognosis could not be conducted. Fifth, our analysis of UF^net^ intensity was based on fixed early time frames (the first 24, 48, and 72 h) and did not account for the dynamic trajectory of fluid removal over time. Future studies employing time-series methods or trajectory-based clustering would offer a more nuanced understanding of how UF^net^ adjustments evolve in response to a patient’s clinical status.

## 5. Conclusions

In conclusion, our study indicates that a UF^net^ intensity between 1.01 and 1.75 mL/kg/h in the first 72 h of CRRT is associated with reduced mortality, irrespective of admission diagnosis. These findings support the implementation of individualized fluid management strategies in CRRT but require prospective validation to establish causality. Future prospective studies are needed to validate optimal UF^net^ thresholds in multicenter ICU cohorts and to incorporate time-series analyses of UF^net^ trajectories for a more comprehensive understanding of dynamic fluid removal responses.

## Supplementary Material

supplement.docx

## Data Availability

The data that support the findings of this study are included in this article and available from the corresponding author upon reasonable request.
