# Supplementary material for: The prognostic impact of net ultrafiltration intensity in critically ill patients receiving continuous renal replacement therapy: a multivariable and propensity-matched analysis
Source: Ren Fail. 2025 Jul 29;47(1):2537810. doi: 10.1080/0886022X.2025.2537810 (PMC12308870; doi:10.1080/0886022X.2025.2537810)

Table S1 Results of Univariable and Multivariable Cox-proportional hazards of the UF^net^ intensity and 28-day mortality

| Variables | Univariable Models | | | Multivariable Models | |
| --- | --- | --- | --- | --- | --- |
|  | HR (95%CI) | P |  | HR (95%CI) | P |
| **UF^net^ intensity** |  |  |  |  |  |
| < 1.01 mL/kg/h | 1.35 (1.06 ~ 1.72) | **0.016** |  | 1.65 (1.33 ~ 2.04) | **<0.001** |
| 1.01-1.75 mL/kg/h | 1.00 (Reference) | / |  | 1.00 (Reference) | / |
| > 1.75 mL/kg/h | 1.31 (1.03 ~ 1.65) | **0.026** |  | 1.24 (1.01 ~ 1.53) | **0.042** |
| **Demographic characteristics** |  |  |  |  |  |
| Age (years) | 1.03 (1.02 ~ 1.03) | **<0.001** |  | 1.12 (1.05 ~ 1.18) | **<0.001** |
| Male sex | 0.86 (0.69 ~ 1.07) | 0.180 |  | 1.66 (1.33 ~ 2.08) | **<0.001** |
| Weight (kg) | 0.81 (0.75 ~ 0.89) | **<0.001** |  | 0.83 (0.76 ~ 0.91) | **<0.001** |
| **Pre-admission kidney function** |  |  |  |  |  |
| Baseline serum creatinine (mg/dl) | 1.00 (1.00 ~ 1.00) | 0.052 |  | 16.04 (2.15 ~ 119.51) | 0.111 |
| **Before CRRT initiation** |  |  |  |  |  |
| Mean arterial pressure (mmHg) | 0.99 (0.98 ~ 0.99) | **0.002** |  | 0.79 (0.65 ~ 0.96) | **0.021** |
| APACHE II score (per 10%) | 0.43 (0.22 ~ 0.81) | **0.009** |  | 0.63 (0.45 ~ 0.90) | **0.012** |
| FO >5% before CRRT | 0.98 (0.76 ~ 1.27) | 0.877 |  | 0.67 (0.54 ~ 0.83) | **<0.001** |
| Interval from admission to CRRT (per 1 day increase) | 1.04 (0.25 ~ 4.33) | 0.961 |  | 1.35 (1.08 ~ 1.70) | **0.009** |
| **Diagnosis** |  |  |  |  |  |
| Trauma | 1.00 (Reference) | / |  | 1.00 (Reference) | / |
| Pneumonia | 1.44 (0.74 ~ 2.81) | 0.288 |  | 0.67 (0.50 ~ 0.89) | **0.006** |
| Respiratory-not pneumonia | 3.03 (1.50 ~ 6.09) | **0.002** |  | 2.86 (1.17 ~ 6.96) | **0.021** |
| Sepsis | 2.35 (1.47 ~ 3.76) | **<0.001** |  | 0.70 (0.55 ~ 0.91) | **0.007** |
| Other medical | 2.20 (1.20 ~ 4.04) | **0.011** |  | 0.99 (0.98 ~ 0.99) | **0.001** |
| Neurology | 1.36 (0.73 ~ 2.55) | 0.337 |  | 1.33 (0.80 ~ 2.23) | 0.274 |
| Digestive | 1.23 (0.76 ~ 1.99) | 0.392 |  | 2.86 (1.64 ~ 4.99) | **<0.001** |
| Cardiovascular | 2.70 (1.59 ~ 4.59) | **<0.001** |  | 2.24 (1.81 ~ 2.76) | **<0.001** |
| Cardiothoracic surgery | 0.63 (0.35 ~ 1.12) | 0.115 |  | 2.48 (1.61 ~ 3.82) | **<0.001** |
| Haematology | 3.94 (1.73 ~ 8.97) | **0.001** |  | 1.21 (0.76 ~ 1.92) | 0.417 |

UF^net^: net ultrafiltration, APACHE II: Acute Physiology and Chronic Health Evaluation, FO: fluid overload, HR: Hazards Ratio, CI: Confidence Interval

Table S2 The association of UF^net^ intensity and 60-days mortality.

|  | Model 1 | |  | Model 2 | |  | Model 3 | | Model 4 | | |
| --- | --- | --- | --- | --- | --- | --- | --- | --- | --- | --- | --- |
|  | HR (95%CI) | *P* |  | HR (95%CI) | *P* |  | HR (95%CI) | *P* |  | HR (95%CI) | *P* |
| UF^net^ intensity (ml/kg/h) |  |  |  |  |  |  |  |  |  |  |  |
| > 1.75 mL/kg/h | 1.31 (1.06 ~ 1.62) | **0.014** |  | 1.17 (0.93 ~ 1.47) | 0.173 |  | 1.17 (0.93 ~ 1.49) | 0.184 |  | 1.32 (1.09 ~ 1.59) | **0.005** |
| 1.01-1.75 mL/kg/h | 1.00 (Reference) | **/** |  | 1.00 (Reference) | **/** |  | 1.00 (Reference) | **/** |  | 1.00 (Reference) | **/** |
| < 1.01 mL/kg/h | 1.31 (1.05 ~ 1.63) | **0.019** |  | 1.54 (1.23 ~ 1.94) | **<.001** |  | 1.45 (1.14 ~ 1.84) | **0.002** |  | 1.51 (1.24 ~ 1.84) | **<0.001** |

HR: Hazard Ratio, CI: Confidence Interval

Model 1: Crude

Model 2: Adjust: age, sex, weight

Model 3: Adjust: age, sex, weight, Creatinine, APACHE II score, Map, FO>5%

Model 4: Adjust: age, sex, weight, Creatinine, APACHE II score, Map, FO>5%, diagnosis, interval from admission to CRRT

Table S3 The association of UF^net^ intensity and 90-days mortality.

|  | Model 1 | |  | Model 2 | |  | Model 3 | | Model 4 | | |
| --- | --- | --- | --- | --- | --- | --- | --- | --- | --- | --- | --- |
|  | HR (95%CI) | *P* |  | HR (95%CI) | *P* |  | HR (95%CI) | *P* |  | HR (95%CI) | *P* |
| UF^net^ intensity (ml/kg/h) |  |  |  |  |  |  |  |  |  |  |  |
| > 1.75 mL/kg/h | 1.29 (1.05 ~ 1.59) | **0.016** |  | 1.14 (0.92 ~ 1.42) | 0.232 |  | 1.15 (0.91 ~ 1.45) | 0.235 |  | 1.28 (1.06 ~ 1.54) | **0.009** |
| 1.01-1.75 mL/kg/h | 1.00 (Reference) | **/** |  | 1.00 (Reference) | **/** |  | 1.00 (Reference) | **/** |  | 1.00 (Reference) | **/** |
| < 1.01 mL/kg/h | 1.25 (1.01 ~ 1.55) | **0.044** |  | 1.48 (1.19 ~ 1.84) | **<0.001** |  | 1.40 (1.11 ~ 1.77) | **0.005** |  | 1.44 (1.18 ~ 1.75) | **<0.001** |

HR: Hazard Ratio, CI: Confidence Interval

Model 1: Crude

Model 2: Adjust: age, sex, weight

Model 3: Adjust: age, sex, weight, Creatinine, APACHE II score, Map, FO>5%

Model 4: Adjust: age, sex, weight, Creatinine, APACHE II score, Map, FO>5%, diagnosis, interval from admission to CRRT

Table S4 Clinical data before and after propensity score methods.

| Variable | Before PSM | | | | | After PSM | | | | |
| --- | --- | --- | --- | --- | --- | --- | --- | --- | --- | --- |
|  | ＜1.01 mL/kg/h  (n=193) | 1.01-1.75 mL/kg/h (n=277) | ＞1.75mL/kg/h  (n=213) | *P* | SMD | ＜1.01 mL/kg/h  (n=101) | 1.01-1.75 mL/kg/h (n=101) | ＞1.75mL/kg/h  (n=101) | *P* | SMD |
| Age, Mean±SD | 54.47±16.63 | 54.87±16.43 | 58.05±16.51 | 0.048 | 0.144 | 55.60±15.62 | 53.52±16.44 | 55.60±15.48 | 0.561 | 0.087 |
| sex, *n* (%) |  |  |  | <0.001 | 0.220 |  |  |  | 0.486 | 0.100 |
| Male | 152 (78.76) | 220 (79.42) | 138 (64.79) |  |  | 78 (77.23) | 84 (83.17) | 78 (77.23) |  |  |
| Female | 41 (21.24) | 57 (20.58) | 75 (35.21) |  |  | 23 (22.77) | 17 (16.83) | 23 (22.77) |  |  |
| Weight, Mean±SD | 71.41±14.85 | 70.08±14.43 | 60.31±12.96 | <0.001 | 0.533 | 67.36±11.03 | 67.79±10.33 | 68.10±10.93 | 0.885 | 0.046 |
| BMI, Mean±SD | 25.55±5.46 | 25.19±4.30 | 22.33±4.08 | <0.001 | 0.476 | 24.50±3.69 | 24.40±3.38 | 24.64±3.59 | 0.889 | 0.045 |
| Intervals from ICU admission to CRRT, M (Q_1_, Q_3_) | 1.04 (0.38, 3.00) | 0.94 (0.29, 4.00) | 1.27 (0.33, 4.00) | 0.417 | 0.012 | 1.42 (0.38, 5.00) | 1.08 (0.42, 4.00) | 1.17 (0.33, 3.00) | 0.701 | 0.118 |
| ICU admission diagnosis, n (%) |  |  |  | 0.982 | 0.189 |  |  |  | 0.998 | 0.250 |
| Cardiothoracic surgery | 33 (17.10) | 32 (11.55) | 25 (11.74) |  |  | 12 (11.88) | 17 (16.83) | 14 (13.86) |  |  |
| Sepsis | 52 (26.94) | 76 (27.44) | 52 (24.41) |  |  | 27 (26.73) | 25 (24.75) | 24 (23.76) |  |  |
| Neurology | 11 (5.70) | 12 (4.33) | 12 (5.63) |  |  | 4 (3.96) | 3 (2.97) | 5 (4.95) |  |  |
| Trauma | 13 (6.74) | 19 (6.86) | 15 (7.04) |  |  | 7 (6.93) | 8 (7.92) | 6 (5.94) |  |  |
| Respiratory-not pneumonia | 4 (2.07) | 7 (2.53) | 4 (1.88) |  |  | 3 (2.97) | 2 (1.98) | 2 (1.98) |  |  |
| Digestive | 52 (26.94) | 83 (29.96) | 62 (29.11) |  |  | 31 (30.69) | 30 (29.70) | 31 (30.69) |  |  |
| Other medical | 7 (3.63) | 10 (3.61) | 13 (6.10) |  |  | 3 (2.97) | 5 (4.95) | 4 (3.96) |  |  |
| Cardiovascular | 13 (6.74) | 23 (8.30) | 19 (8.92) |  |  | 10 (9.90) | 10 (9.90) | 10 (9.90) |  |  |
| Pneumonia | 6 (3.11) | 11 (3.97) | 9 (4.23) |  |  | 4 (3.96) | 1 (0.99) | 4 (3.96) |  |  |
| Haematology | 2 (1.04) | 4 (1.44) | 2 (0.94) |  |  | 0 (0.00) | 0 (0.00) | 1 (0.99) |  |  |
| Creatinine, M (Q_1_, Q_3_) | 224.00 (152.00, 362.00) | 281.00 (182.00, 401.00) | 245.00 (154.00, 377.00) | 0.005 | 0.208 | 252.00 (182.00, 386.00) | 273.00 (200.00, 373.00) | 253.00 (153.00, 370.00) | 0.488 | 0.069 |
| PLT, M (Q_1_, Q_3_) | 94.00 (53.00, 167.00) | 95.00 (53.00, 154.00) | 77.00 (43.00, 139.00) | 0.032 | 0.124 | 94.00 (48.00, 173.00) | 104.00 (62.00, 151.00) | 84.00 (43.00, 150.00) | 0.367 | 0.063 |
| NT-proBNP, M (Q_1_, Q_3_) | 3326.00 (993.00, 9412.00) | 4198.00 (1260.25, 11262.75) | 5423.50 (1817.75, 15215.00) | 0.002 | 0.168 | 4327.00 (1290.00, 10286.00) | 4105.00 (1223.00, 13188.00) | 5439.00 (1824.00, 13537.00) | 0.623 | 0.046 |
| Lactate, M (Q_1_, Q_3_) | 2.30 (1.50, 4.30) | 2.00 (1.40, 3.40) | 2.00 (1.30, 3.10) | 0.009 | 0.158 | 2.00 (1.50, 3.40) | 2.00 (1.50, 2.90) | 2.00 (1.40, 3.70) | 0.874 | 0.064 |
| APACHE II score, M (Q_1_, Q_3_) | 21.00 (14.00, 27.00) | 22.00 (16.00, 28.00) | 21.00 (15.00, 27.00) | 0.237 | 0.107 | 20.00 (14.00, 26.00) | 21.00 (16.00, 27.00) | 21.00 (14.00, 27.00) | 0.857 | 0.058 |
| Map, Mean±SD | 86.17±15.44 | 85.97±14.05 | 86.70±14.48 | 0.857 | 0.033 | 86.92±16.51 | 86.78±13.48 | 88.23±14.66 | 0.747 | 0.066 |

UF^net^: net ultrafiltration, CRRT: continuous renal replacement therapy, BMI: body mass index, ICU: intensive care unit, PLT: platelet, NT-proBNP: N-terminal pro-brain natriuretic peptide, APACHE II: Acute Physiology and Chronic Health Evaluation, MAP: mean arteial pressure.

Table S5 Patient characteristics, outcomes and CRRT related data by admission diagnosis category.

^*^Median (interquartile range).

| **Admission diagnosis category** | **n** | **Age, years (IQR)*** | **Weight, kg (IQR)*** | **Males, n (%)** | **Time to CRRT, days (IQR)*** | **ICU LOS, days (IQR)*** | **CRRT time, hours (IQR)^*^** | **Mechanical ventilation treatment days (IQR)*** | **Died ICU, n (%)** |
| --- | --- | --- | --- | --- | --- | --- | --- | --- | --- |
| Cardiovascular | 55 | 63 (47 – 76) | 62 (55 – 70) | 34 (61.8%) | 0.96 (0.46 – 3) | 14 (8 – 21) | 117 (92 – 144) | 10 (5 – 16) | 46 (83.6%) |
| Cardiothoracic surgery | 90 | 56 (48.75 – 68) | 65 (55 – 75) | 64 (71.1%) | 1.88 (0.88 – 4) | 14 (10 – 19.25) | 126.5 (107 – 147.25) | 11 (7 – 16.25) | 25 (27.8%) |
| Neurology | 35 | 56 (41 – 71) | 73 (60 – 80) | 29 (82.9%) | 1.19 (0.45 – 9.75) | 26 (15 – 45) | 125 (93 – 142) | 18 (9 – 28) | 22 (62.9%) |
| Respiratory - not pneumonia | 15 | 69 (63 – 76) | 60 (55 – 66.5) | 11 (73.3%) | 1.54 (0.21 – 8) | 18 (12 – 22) | 131 (113 – 149) | 15 (11 – 20) | 13 (86.7%) |
| Pneumonia | 26 | 63.5 (51 – 74.75) | 64.5 (55 – 75) | 20 (76.9%) | 2.15 (0.45 – 8.25) | 23.5 (11.75 – 36.75) | 143.5 (118.75 – 148.75) | 20 (9 – 30) | 20 (76.9%) |
| Haematological | 8 | 61.5 (31 – 75.75) | 63.5 (56.25 – 81) | 5 (62.5%) | 1.19 (0 – 3.52) | 10 (7.75 – 14) | 130 (93.5 – 152.5) | 7 (4.5 – 13) | 5 (62.5%) |
| Digestive | 197 | 49 (39.5 – 60) | 70 (64.75 – 80) | 151 (76.6%) | 0.79 (0.22 – 2.41) | 20 (14 – 32) | 139 (117 – 147) | 15 (9 – 24) | 111 (56.3%) |
| Sepsis/infection - not pneumonia | 180 | 63(53 – 73) | 65(55 – 75) | 139 (77.2%) | 1.17 (0.33 – 6) | 16(11 – 27) | 132.5 (109.25 – 149) | 13.5 (7.25 – 22.75) | 139 (77.2%) |
| Trauma | 47 | 54.84 (50.2 to 59.5) | 91.1 (85.81 to 96.4) | 38 (80.9%) | 1.7 (0.7 to 5.5) | 13.7 (8 to 21) | 133 (112 – 144) | 15 (8.5 – 26) | 27 (57.4%) |
| Other medical | 30 | 55.5 (47.25 – 68) | 66.5 (48.75 – 75.5) | 19 (63.3%) | 0.58 (0.17 – 4.25) | 15.5 (10 – 25) | 129.5 (114.75 – 144.25) | 13.5 (6.75 – 21) | 22 (62.91%) |

| **Variable** | **Hazard Ratio** | **p** | **Variable** | **Hazard Ratio** | **p** |
| --- | --- | --- | --- | --- | --- |
| ***Cardiothoracic surgery*** |  |  | ***Pneumonia*** |  |  |
| UF^net^ in first 72 hours (per mL/kg/hr) | 1.02 (0.60 to 1.74) | 0.94 | UF^net^ in first 72 hours (per mL/kg/hr) | 0.84 (0.36 to 1.95) | 0.68 |
| Weight (per 10kg) | 1.05 (0.69 to 1.58) | 0.83 | Weight (per 10kg) | 0.80 (0.49 to 1.29) | 0.80 |
| APACHE III risk of death (per 10%) | 0.96 (0.60 to 1.53) | 0.87 | APACHE III risk of death (per 10%) | 1.62 (0.81 to 3.26) | 0.17 |
| Age (per 10 years) | 1.26 (0.94 to 1.68) | 0.13 | Age (per 10 years) | 1.11 (0.78 to 1.59) | 0.56 |
| Male sex | 0.83 (0.31 to 2.26) | 0.72 | Male sex | 0.96 (0.23 to 3.99) | 0.95 |
| ***Trauma*** |  |  | ***Respiratory - not pneumonia*** |  |  |
| UF^net^ in first 72 hours (per mL/kg/hr) | 0.88 (0.53 to 1.48) | 0.63 | UF^net^ in first 72 hours (per mL/kg/hr) | 0.34 (0.09 to 1.23) | 0.10 |
| Weight (per 10kg) | 0.93 (0.60 to 1.47) | 0.93 | Weight (per 10kg) | 0.14 (0.04 to 0.48) | 0.002* |
| APACHE III risk of death (per 10%) | 1.97 (1.08 to 3.59) | 0.027* | APACHE III risk of death (per 10%) | 16.89 (2.38 to 120.04) | 0.005* |
| Age (per 10 years) | 1.34 (1.02 to 1.75) | 0.034* | Age (per 10 years) | 0.87 (0.54 to 1.40) | 0.57 |
| Male sex | 1.67 (0.55 to 5.12) | 0.37 | Male sex | 0.11 (0.01 to 0.92) | 0.041* |
| ***Cardiovascular*** |  |  | ***Haematological*** |  |  |
| UF^net^ in first 72 hours (per mL/kg/hr) | 0.97 (0.59 to 1.59) | 0.90 | UF^net^ in first 72 hours (per mL/kg/hr) | 6.60 (0.10 to 434.23) | 0.38 |
| Weight (per 10kg) | 0.76 (0.54 to 1.08) | 0.13 | Weight (per 10kg) | 0.88 (0.21 to 2.64) | 0.86 |
| APACHE III risk of death (per 10%) | 1.08 (071 to 1.65) | 0.72 | APACHE III risk of death (per 10%) | 1.16 (0.22 to 6.20) | 0.86 |
| Age (per 10 years) | 0.97 (0.83 to 1.14) | 0.72 | Age (per 10 years) | 1.93 (0.65 to 5.73) | 0.23 |
| Male sex | 0.72 (0.26 to 1.46) | 0.36 | Male sex | 0.01 (0.00 to 2.71) | 0.11 |
| ***Sepsis*** |  |  | ***Other medical*** |  |  |
| UF^net^ in first 72 hours (per mL/kg/hr) | 0.87 (0.67 to 1.13) | 0.28 | UF^net^ in first 72 hours (per mL/kg/hr) | 1.15 (0.66 to 2.00) | 0.63 |
| Weight (per 10kg) | 0.90 (0.75 to 1.08) | 0.27 | Weight (per 10kg) | 0.78 (0.54 to 1.12) | 0.18 |
| APACHE III risk of death (per 10%) | 1.04 (0.87 to 1.25) | 0.65 | APACHE III risk of death (per 10%) | 1.10 (0.59 to 2.04) | 0.77 |
| Age (per 10 years) | 1.24 (1.11 to 1.38) | <0.0001* | Age (per 10 years) | 1.17 (0.85 to 1.60) | 0.34 |
| Male sex | 0.91 (0.60 to 1.38) | 0.66 | Male sex | 0.71 (0.27 to 1.85) | 0.48 |
| ***Digestive*** |  |  | ***Neurology*** |  |  |
| UF^net^ in first 72 hours (per mL/kg/hr) | 0.87 (0.67 to 1.13) | 0.31 | UF^net^ in first 72 hours (per mL/kg/hr) | 0.76 (0.38 to 1.51) | 0.43 |
| Weight (per 10kg) | 0.88 (0.72 to 1.08) | 0.21 | Weight (per 10kg) | 0.59 (0.34 to 1.02) | 0.06 |
| APACHE III risk of death (per 10%) | 1.04 (0.84 to 1.28) | 0.73 | APACHE III risk of death (per 10%) | 0.81 (0.55 to 1.20) | 0.29 |
| Age (per 10 years) | 1.26 (1.11 to 1.42) | 0.001* | Age (per 10 years) | 1.27 (0.98 to 1.64) | 0.08 |
| Male sex | 0.93 (0.59 to 1.44) | 0.73 | Male sex | 0.61 (0.17 to 2.22) | 0.45 |

Table S6 Cox proportional hazards model showing UF_NET_ by admission diagnosis.

Figure S1. Kaplan–Meier survival plots with the log-rank test by different UF^net^ intensity category in the first 72h.


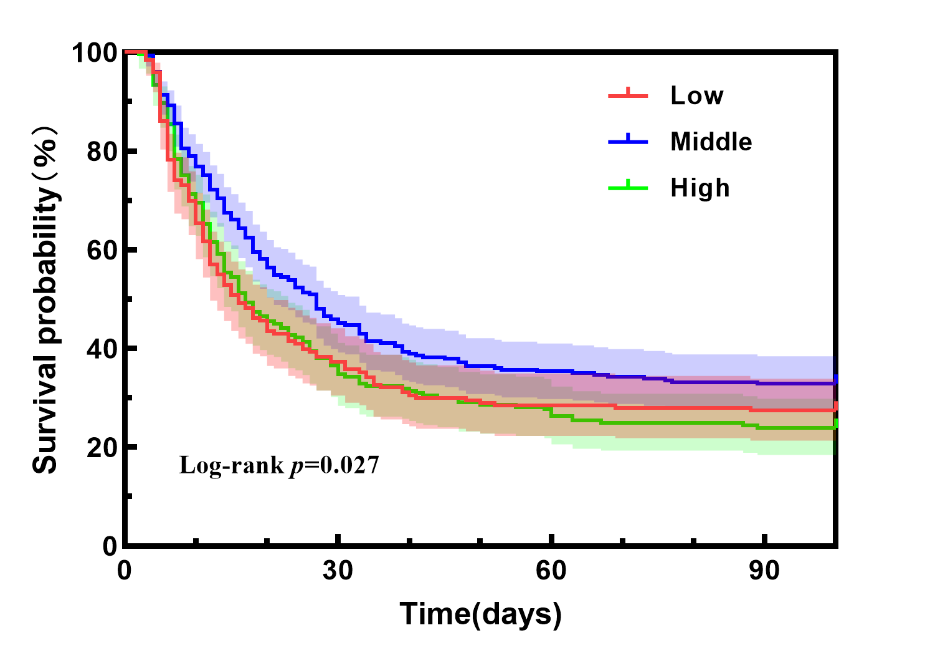


Figure S2. Kaplan–Meier survival plots with the log-rank test by different UF^net^ intensity category in the first 24h.


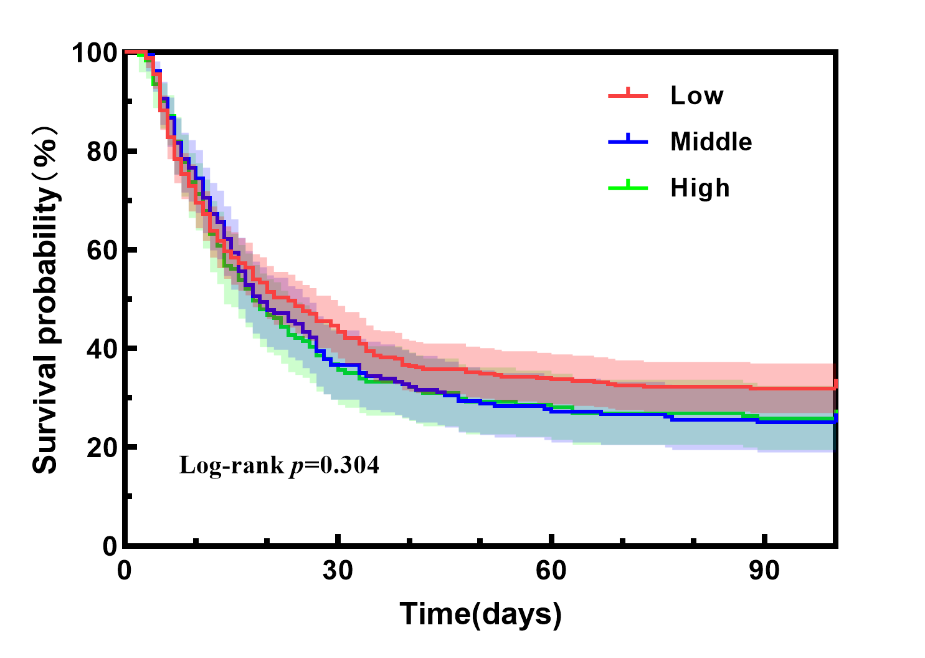


Figure S3. Kaplan–Meier survival plots with the log-rank test by different UF^net^ intensity category in the first 48h.


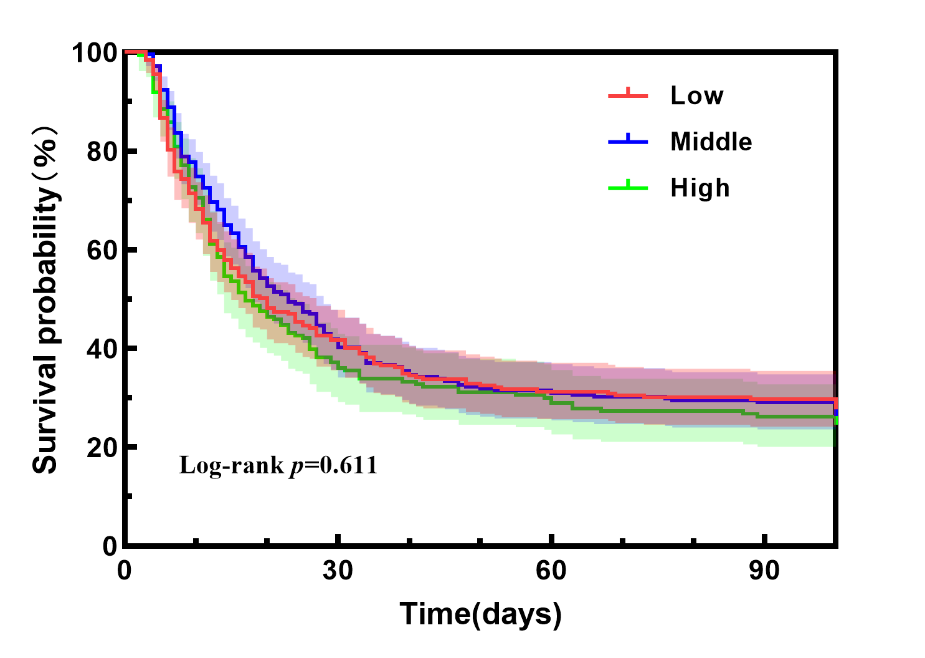


Figure S4. Kaplan–Meier survival plots with the log-rank test by different UFnet intensity category in the first 72h after Propensity Score Matching.


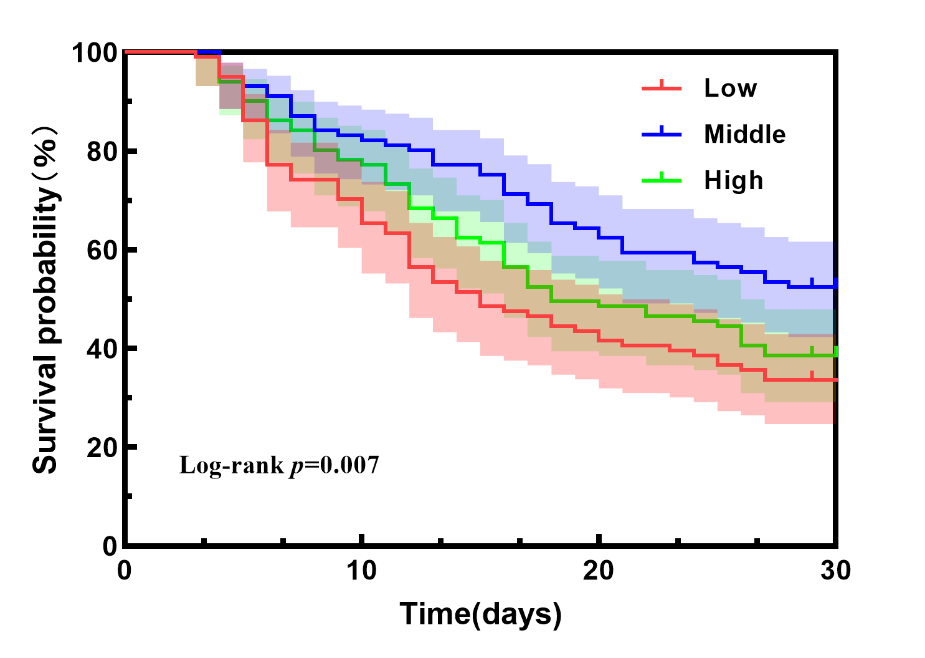


Figure S5. UF^net^ intensity in the first 72h by admission diagnosis.


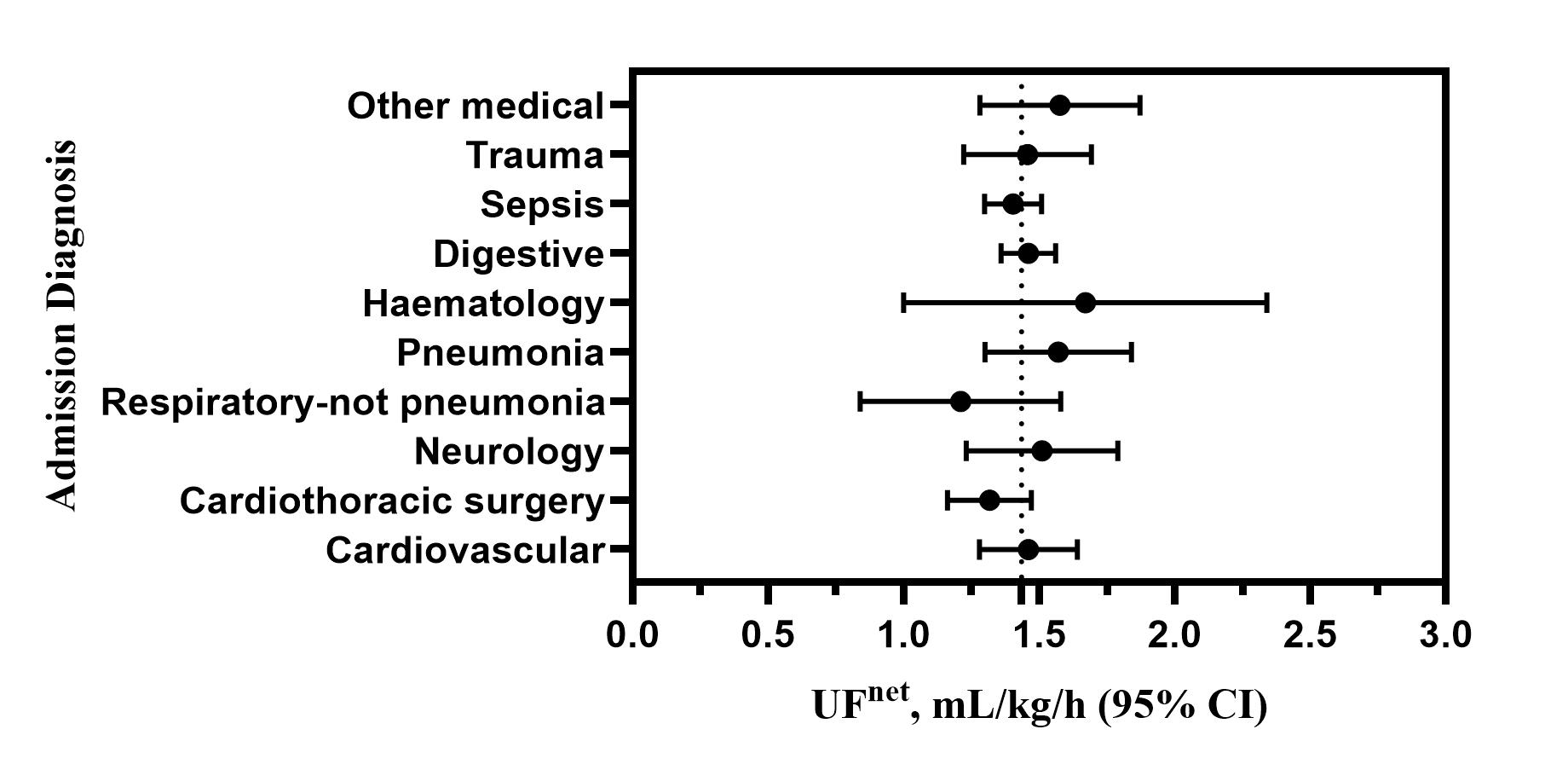


Figure. S6. Cox models for each admission diagnosis subgroup, hazard of UF^net^ intensity Hazard ratios with 95% confidence intervals are shown for each admission diagnosis.


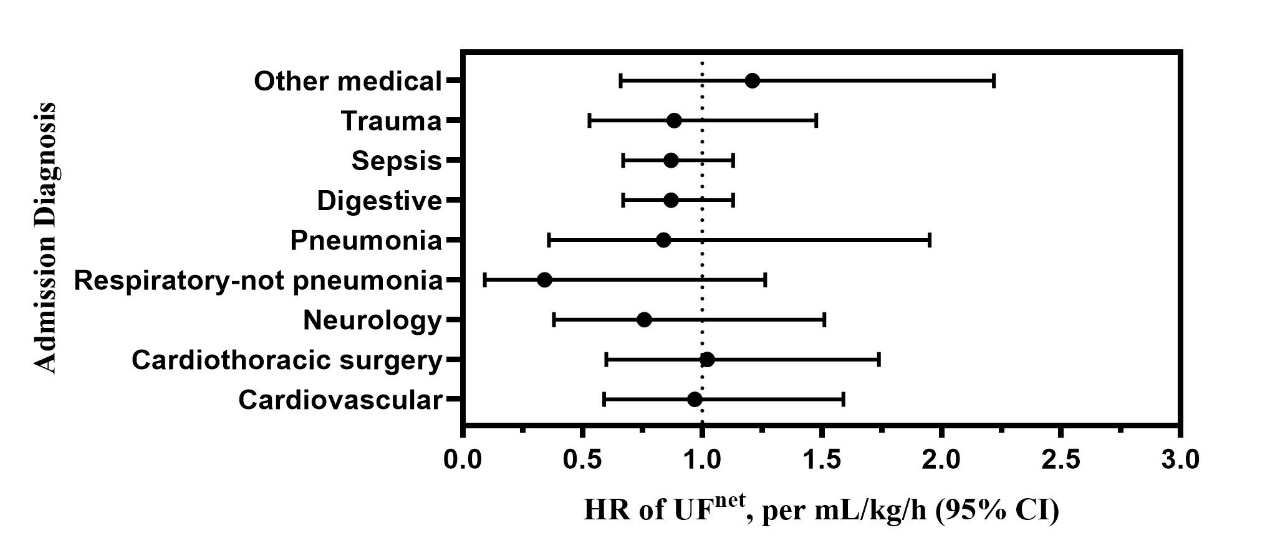

Supplement: supplement.docx [file IRNF_A_2537810_SM0160.docx]
